# Clinical Decision Support System for Diabetic Patients by Predicting Type 2 Diabetes Using Machine Learning Algorithms

**DOI:** 10.1155/2023/6992441

**Published:** 2023-05-30

**Authors:** Rakibul Islam, Azrin Sultana, Md. Nuruzzaman Tuhin, Md. Sazzad Hossain Saikat, Mohammad Rashedul Islam

**Affiliations:** ^1^Department of Computer Science, American International University-Bangladesh, Dhaka 1229, Bangladesh; ^2^Department of Research & Training Monitoring, Bangladesh College of Physicians and Surgeons, Dhaka 1212, Bangladesh; ^3^Department of Health Informatics, Bangladesh University of Health Sciences, Dhaka 1216, Bangladesh

## Abstract

Diabetes is one of the most serious chronic diseases that result in high blood sugar levels. Early prediction can significantly diminish the potential jeopardy and severity of diabetes. In this study, different machine learning (ML) algorithms were applied to predict whether an unknown sample had diabetes or not. However, the main significance of this research was to provide a clinical decision support system (CDSS) by predicting type 2 diabetes using different ML algorithms. For the research purpose, the publicly available Pima Indian Diabetes (PID) dataset was used. Data preprocessing, K-fold cross-validation, hyperparameter tuning, and various ML classifiers such as K-nearest neighbor (KNN), decision tree (DT), random forest (RF), Naïve Bayes (NB), support vector machine (SVM), and histogram-based gradient boosting (HBGB) were used. Several scaling methods were also used to improve the accuracy of the result. For further research, a rule-based approach was used to escalate the effectiveness of the system. After that, the accuracy of DT and HBGB was above 90%. Based on this result, the CDSS was implemented where users can give the required input parameters through a web-based user interface to get decision support with some analytical results for the individual patient. The CDSS, which was implemented, will be beneficial for physicians and patients to make decisions about diabetes diagnosis and offer real-time analysis-based suggestions to improve medical quality. For future work, if daily data of a diabetic patient can be put together, then a better clinical support system can be implemented for daily decision support for patients worldwide.

## 1. Introduction

A CDSS can be a blessing in the field of chronic diseases like diabetes. The capacity, complexity, and dynamic behavior of clinical info are a challenge for doctors and other health professionals. CDSS seeks to favor the physicians as well as the patients by providingreal-time feedback regarding health conditions [1].

Diabetes is a chronic metabolic condition marked by a recurrent rise in blood glucose levels. It is a global health priority that affects 463 million people, or one out of every eleven adults. This figure is anticipated to grow to 578 million by 2030 [2]. Diabetes is caused by several different pathogenic mechanisms. These can range from autoimmune death of pancreatic beta cells, resulting in insulin shortage, to anomalies that lead to insulin resistance. Due to the poor impact of insulin on target tissues, diabetes produces abnormalities in glucose, lipid, and protein metabolism. Insulin deficiency happens when the body does not make enough insulin and/or when tissues do not respond well enough to insulin at one or more points along the complicated path of hormone action. In many patients, reduced insulin secretion and impaired insulin movement coexist, and it is tough to inform which condition, if either, is the essential supply of hyperglycemia [3]. Diabetes is a category of metabolic disorders marked by hyperglycemia caused by problems with insulin secretion, insulin action, or both. Long-term injury, dysfunction, and breakdown of various organs, primarily the eyes, kidneys, nerves, heart, and blood vessels, have been linked. The patient's health conditions should be regularly monitored to prevent these complications. To eliminate health hazards, early prediction of diabetes can be very beneficial as well as CDSS will help patients to perform continuous observation of different parameters that control insulin levels.

Over the most recent twenty years, the advancement of patient management conformity has been altogether expanded in healthcare [4–7]. In this research, our ultimate goal was to propose a CDSS for diabetes patients and clinicians, so accurately predicting diabetes was the first goal. To predict diabetes, different ML classification algorithms such as KNN, DT, RF, HBGB, and NB were used. Patients will have access to a user interface to predict diabetes based on input parameters. With a comparative analysis of the input parameters, decision support will be offered depending on the results of the prediction system.

### 1.1. Contributions of the Proposed Work

The main contributions of this study are as follows:

- The proposed system is provided with a diabetes prediction system as well as a decision support system which will give a graphical analysis to the patients.
- In this study, various scaling methods have been applied to different ML algorithms that have provided different levels of accuracy.
- A rule-based technique was applied to the dataset to improve the accuracy.
- Histogram-based gradient boosting algorithm achieved the highest accuracy of 92.2%.

The remaining portion of the discussion of the research is structured as follows. The next part of this section discusses the related work of the different researchers in the same field.  Section 2 covers methodology, model diagram of the system, dataset description, and preprocessing.  Section 3 is about results, analysis, and discussion, and Section 4 covers the conclusion part of the research work.

### 1.2. Related Work

The goal of Kopitar et al.'s prediction model [8] was to predict type 2 diabetes at an early stage using ML approaches and to examine if ML-based techniques gave any benefit in the early detection of impaired fasting glucose and fasting plasma glucose level readings. This study's data came from 10 hospitals in Slovenia with 3723 participants. In the beginning, there were 111 variables, but only 59 were used because the others had missing values. The variables were then divided into four categories. For this model, five ML techniques were used: LR, Glmnet, XGBoost, RF, and LightGBM. The predictive model was validated using root mean square error. AUC and area under the precision-recall curve were employed to evaluate the system because the dataset was imbalanced. XGBoost had 88.1% accuracy.

Alaa Khaleel and Al-Bakry [9] utilized the PID dataset and three supervised ML algorithms, including logistic regression (LR), NB, and KNN, to predict diabetes. The dataset was preprocessed with the MinMax scaler to get a better accuracy value. The model was partitioned into a 7 : 3 ratio for training and testing purposes. LR algorithms are hailed as the best classifier technique for this suggested system since their precision is superior to other classification algorithms.

The authors of [10] suggested an IoT and ML system that analyzes blood sugar and other essential indicators to identify diabetes early and improve diabetes management apps that aid in patient monitoring. Four sensors were applied to obtain the essential clinical data. Additionally, a questionnaire was also employed to collect data. After the dataset was prepared, four distinct ML algorithms were implemented. The PID dataset was utilized to evaluate the algorithms' accuracy with their collected dataset. A web-based diabetes management strategy was also proposed in this research.

Kaur and Kumari [11] developed a model utilizing the PID dataset and five distinct ML methods, including KNN, linear kernel SVM, SVM radial basis kernel, artificial neural network, and multifactor dimensionality reduction. Via the Boruta wrapper technique, significant features of the dataset were chosen. All models were tested using several criteria, including accuracy, *F*1 score, recall, precision, and AUC. SVM-linear performed much better than other models.

Rghioui et al. [12] devised a method for monitoring the blood glucose level of diabetic patients based on ML methods. The proposed system develops an algorithm based on ML techniques and big data that can analyze the data of diabetes patients and send an alert in the event of an emergency. The data were sent to the server using 5G technology. The architecture of 5G technology consists of sensors, wearable devices, a smartphone application, and a server with a database. There were portable sensors that could monitor the patient's blood glucose level, physical activity, and temperature and transfer the data to the base station for analysis by ML algorithms through 5G. The WEKA software was utilized for this study. In addition, the proposed method helps diabetes patients forecast their future blood sugar levels.

Deepti and Dilip [13] employed three ML classification methods to predict diabetes, such as DT, SVM, and NB. They also used the PID dataset in their study. Different accuracy measures like *F*-measure, precision, recall, and receiver operating curve were introduced for evaluating the performance of the algorithms. A 10-fold cross-validation method was also implemented in the dataset. The highest accuracy of 76.30% was achieved by the NB method.

In [14], Rajput et al. proposed a cloud-based mobile application framework which has been proposed to help the rural patients to monitor their type 2 diabetes through regular follow-up of daily step counts, physical activity, and daily travel history. They also indicated that lifestyle is one of the main reasons why people get diabetes, so they came up with a plan to help people keep track of their lifestyle and control their diabetes, which will be observed securely by doctors and medical practitioners. Hence, this can improve the interactivity between patients and doctors.

To conclude, the literature review shows that various researchers have made contributions to the diabetes prediction model. In addition, the study indicates that most of the researchers used the PID dataset to predict diabetes using different algorithms. However, the main research gap we observed is that most of the authors have only worked on the predictive model in this domain with this dataset. But this study also proposed a CDSS that offers a web-based system where patients can give inputs and will have some recommendations and comparative analytics graphs according to the output. Additionally, we have used different scaling methods to observe the results from different ML techniques. A rule-based approach was also used for the PID dataset, which has improved the accuracy. Therefore, we consider the proposed methodology to be an invaluable contribution to both this dataset and the healthcare industry as a whole.

## 2. Methodology

### 2.1. Model Diagram

**Figure 1:**
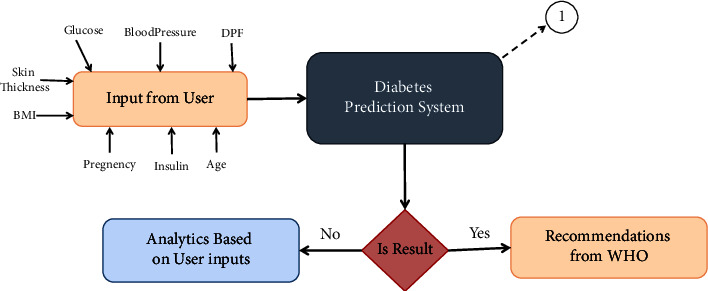
Block diagram of the proposed CDSS.

**Figure 2:**
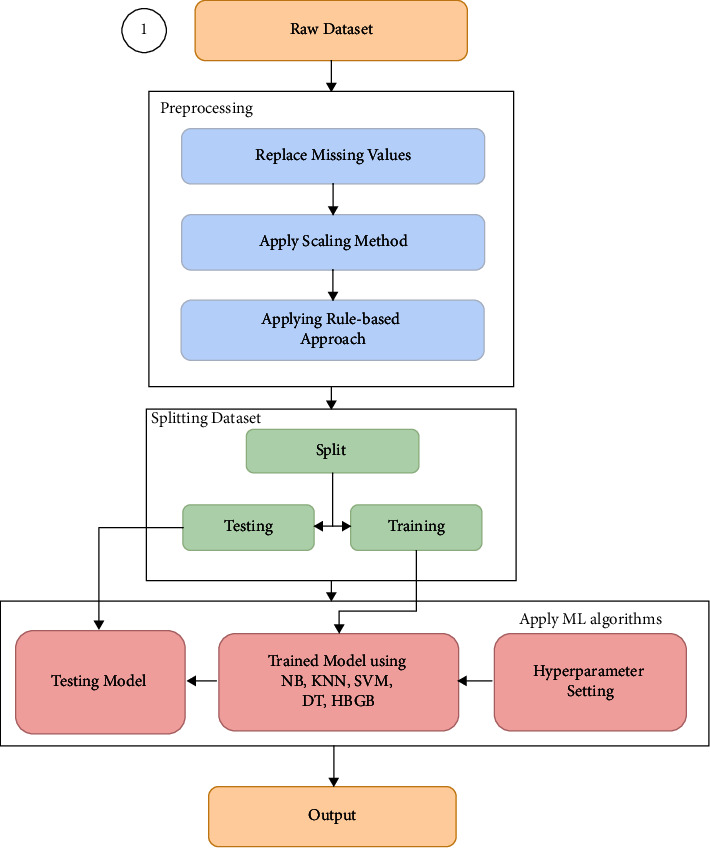
Block diagram of diabetes prediction system.

The proposed system is represented in Figures 1 and 2. As our model works in two stages, the first stage, in Figure 2, refers to the prediction system. The second stage, in Figure 1, denotes the model architecture of the user interface where the user can give the required input value to have some decision support as well as some comparative analysis which is discussed elaborately in the result section. As shown in the block diagram in Figure 2, accumulating the dataset was the preliminary step in the predictive model. The PID dataset was used in this study, which has 768 samples. As the dataset contains missing values, according to the predictive model diagram, the dataset must be preprocessed before going to the splitting and ML algorithm application phase. Removing missing values, applying different scaling methods, and using some rules to the dataset were mainly in the preprocessing part of this architecture. After that, splitting the dataset into training and testing sets was done. Thus, after completing the preprocessing stage, the whole process went to the next phase, where ML algorithms have been applied to the training dataset so that the testing dataset can be applied to get the output of the algorithm. Finally, we got the output result as “yes” or “no.” This obtained result will be used by the second stage in Figure 1. The proposed CDSS will be formed based on the result of the prediction system according to the user input, which is shown in Figure 1, where comparing the result, the user will be able to get recommendations if the user has diabetes or analytics when the user does not have diabetes.

### 2.2. Brief Description of Algorithms Used

#### 2.2.1. K-Nearest Neighbor

When using KNN, the function is approximated initially, and all computation is deferred until the classification is complete. An n-dimensional space is used to store the data for later analysis, and this is where the training samples are stored. KNN is an essential supervised ML method, despite its simplicity. A supervised ML algorithm uses labeled input data to develop a function that can provide an output when fresh unlabeled data are supplied [15–17]. Euclidean distance for two points A (*x*_1_, *y*_1_) and B (*x*_2_, *y*_2_):

(1) $$\begin{array}{c} \sqrt{{\left(x_{2}-x_{1}\right)}^{2}+{\left(Y_{2}-Y_{1}\right)}^{2}}\text{.} \end{array}$$

In KNN, different *k* values create different clusters for prediction. It is recommended to choose larger *k* values. But the standard range for *k* values is 3 to 10.

#### 2.2.2. Decision Tree

Decision trees refer to the group features according to the sorted form of their values. DT is one of the popular classification techniques of ML. There are several branches and nodes in DT. Each node represents a set of attributes that the numerator, the network classification system, must classify [18–20]. The determination of the attribute for the root node for each level is a key difficulty in the DT. Attribute selection is the term for this procedure. There are two widely used attribute selection methods. Finding the highest information gain and the smallest entropy is the main objective of the DT. Entropy determines how a DT chooses to split data. It affects the manner in which a DT generates its boundaries [21]. The formula for calculating the entropy (*E*):

(2) $$\begin{array}{c} E\left(m\right)=\overset{c}{\underset{i=1}{\sum }}p_{i}\log_{2}p_{i}\text{,} \end{array}$$

where *p*_*i*_ = probability of event *i* in class *m*.

Information gain is calculated from the average value of the entropy before and after splitting, depending on the given value. We have the equation as follows:

(3) $$\begin{array}{c} information\;gain=E\left(b\right)-\overset{k}{\underset{j=1}{\sum }}E\left(x,a\right)\text{,} \end{array}$$

where *E*(*b*) = entropy before the split; *K* = total subsets after splitting; and *E*(*x*, *a*) = total number of subsets after splitting.

#### 2.2.3. Random Forest

The random forest (RF) approach is a DT-based ensemble method. By merging numerous overfit evaluators (i.e., DT) into an ensemble learning algorithm, RF helps to minimize the overfitting tendency of the dataset. The relevant classification decision result can be obtained for each DT. According to the concept of minority following the majority, the classification of the sample measured is determined by the voting results of every decision branch of a tree, and the category with the highest votes in all decision trees is picked as the final result [22, 23]. For the discretion of the dataset, we will need the lowest Gini index. For calculating the Gini index,

(4) $$\begin{array}{c} G=1-\overset{c}{\underset{i=1}{\sum }}p_{i}^{2}\text{,} \end{array}$$

where *P*_*i*_ = probabilistic class.

#### 2.2.4. Support Vector Machine

SVM (also known as vector networks) is another method of supervised learning algorithms. Working with classification and regression analysis in ML, SVM performs better. Given a collection of training examples that are individually labeled as belonging to one of two categories, the SVM technique generates a linear model that generates a system that allocates new instances to one of two groups, as it is a nonprobabilistic binary linear classifier in ML [24, 25].

### 2.3. Dataset Description

In this study, the PID dataset is used for implementing the prediction system, as it is a well-known and widely used benchmark dataset for predicting diabetes [26]. This dataset contains 768 samples along with nine attributes. Here eight attributes are independent; they are age, pregnancies, glucose, blood pressure (BP), skin thickness, insulin, BMI, and diabetes pedigree function, and one attribute is dependent. It is also the resultant feature which is represented with binary values 0 and 1. Here 0 is diabetes negative, and 1 is diabetes positive. From those 768 instances, 500 tested diabetes negative, and 268 tested positive.

### 2.4. Dataset Preprocessing

Preprocessing is the key to getting the preferred output from a dataset. The PID dataset has some unnecessary zero values for certain important features. There are a few ways to get rid of these zero values, such as eliminating rows with zero values and exchanging this with mean or median values. In this research, the median values were used to replace the zero values where necessary.  Table 1 represents different parameters of features such as BMI, glucose, insulin, and BP, respectively. The information about zero values, distinct values, the minimum and maximum range of the individual features, and also the mean values is shown in Table 1.

After replacing the zero values with median values, different scaling methods, such as MinMax scaler, standard scaler, MaxAbs scaler, robust scaler, quantile transformer, and power transformer, were applied while working on different ML algorithms. In this research, a set of rules was integrated with the dataset features based on the correlation of different attributes. The training set is used to train the model, and the testing set is used to test the model's correctness. In this research, 80% of the dataset is used to train the model, and 20% of the dataset is used for testing purposes. The most famous K-fold cross-validation technique [27] has been used to eliminate overfitting and make the dataset unbiased. In this research, we used *k* = 5, which means our dataset has been divided randomly into 5 subparts while applying the algorithms.

### 2.5. Accuracy Metrics

In this research, precision, recall, *F*1 score, and accuracy measurements are evaluated. Precision is defined as the anticipated percentage of true positives against total positives. Recall, also known as sensitivity and true positive rate, denotes the percentage of identified positive classes which were actually positive. *F*1 score is the average value of precision and recall. The formula for precision, recall, *F*1 score, and accuracy [28, 29] is as follows:

(5) $$\begin{array}{c} precision\left(P\right)=\frac{TP}{\left(TP+FP\right)}\text{,}\\ recall\left(R\right)=\frac{TP}{\left(TP+FN\right)}\text{,}\\ F1\;score\left(F\right)=\frac{2\left(P*\;R\right)}{\left(P+R\right)}\text{,}\\ accuracy\left(A\right)=\frac{\left(TP+TN\right)}{\left(TP+TN+FP+FN\right)}\text{.} \end{array}$$

If the prediction system predicts a user as diabetes positive and it is actually positive, then it will be denoted by TP, which means true positive. TN represents true negative which means the prediction system predicts a user as diabetes negative, and it is actually negative. FP represents false positive, which means the prediction system predicts a user as diabetes positive and it is actually negative. FN represents a false negative, which means that the prediction system predicts a user as diabetes negative and it is actually positive.

### 2.6. The Working Process of the User Interface for Decision Support

**Figure 3:**
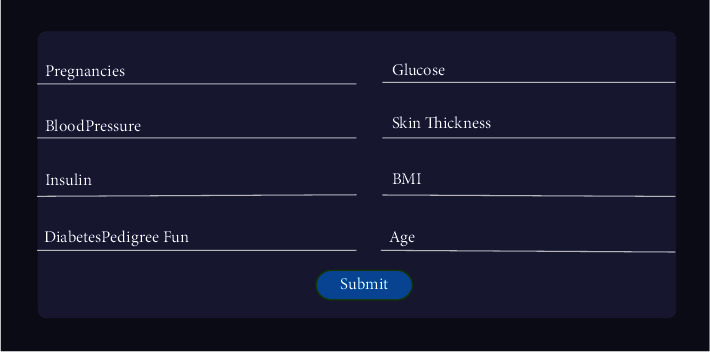
Taking patient's data for new prediction.

As mentioned above, this system is not only going to predict diabetes; hence, decision support will be provided for the patient based on their input values through the web interface shown in Figure 3. These data will be stored and used for prediction. After storing the data collected from the patients, the system will predict the existence of diabetes. If the patient is predicted diabetes positive, then the system will provide some decision support as well as a comparative graph for different parameters in the negative cases.

## 3. Results and Discussion

In this research, several ML approaches were applied for the classification of the PID dataset. The accuracy of the algorithms which were applied to the raw dataset is shown in Table 2.

It can be seen that (from Table 1) the dataset contains zero values in some attributes, such as BMI, glucose, BP, and insulin. But in real life, this cannot be possible. So, the irrelevant zero values are replaced by the mean value of the individual column values. After that, different scaling methods are applied to the dataset to improve the accuracy, and the results are shown in Table 3.

After applying various scaling methods, the comparison of different ML classifier models is evaluated. From the information in Table 3, the KNN model provides the highest accuracy among all other classifiers. The accuracy was 84.02% for the MinMax scaling technique when the value of *k* was 11, and the *p* value was 2 for the KNN algorithm. The lowest accuracy of 73.96% was observed using the DT classification method. On the other hand, it can be seen that HBGB has no impact on the applications of scaling methods. However, different scaling approaches definitely had distinct effects on the classification algorithms, which is helpful in augmenting the model accuracy through a trial-and-error technique. In the final phase, the rule-based approaches were implemented [30–32] to the dataset. This time the zero values were replaced by the median value of the corresponding column. The rules are given in Table 4.

After applying rules, the accuracy was significantly improved compared to the last phase, which is shown in Table 5.

**Figure 4:**
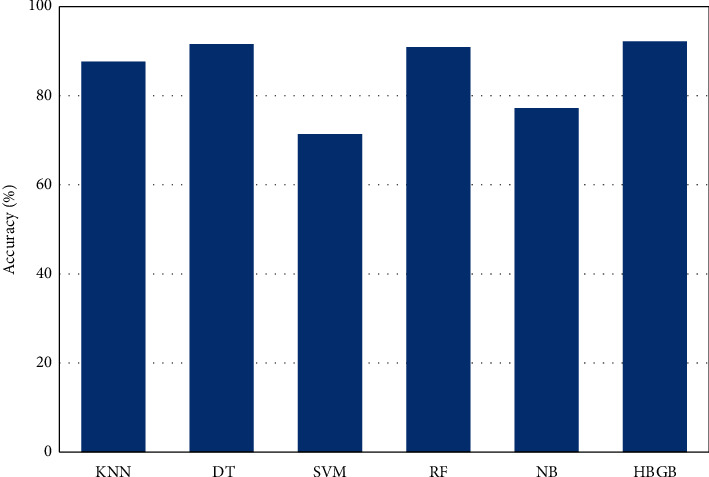
Comparison of accuracy among different algorithms.

**Figure 5:**
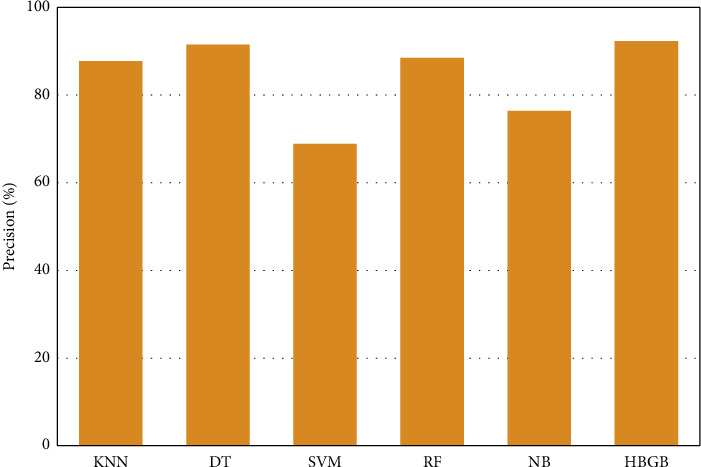
Comparison of precision values among algorithms.

**Figure 6:**
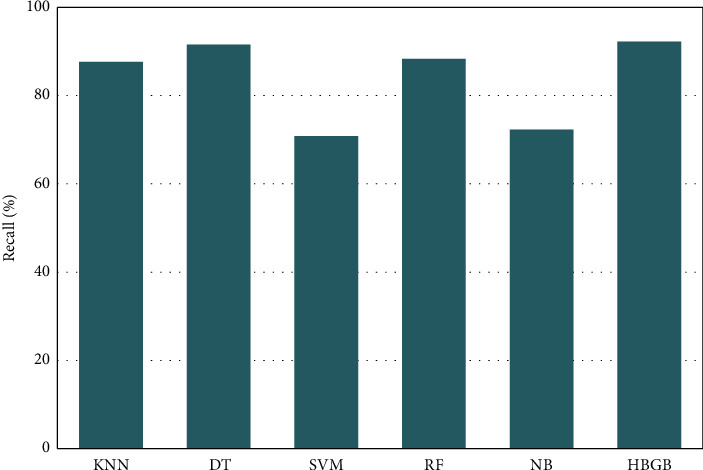
Comparison of recall values among algorithms.

**Figure 7:**
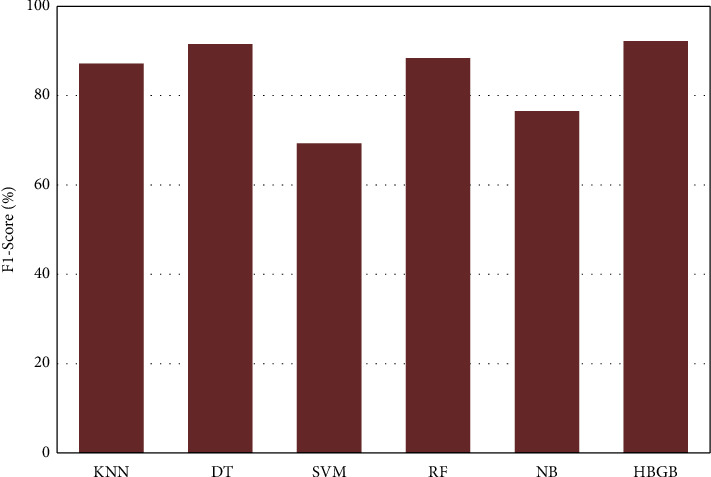
Comparison of *F*1 score among algorithms.

**Figure 8:**
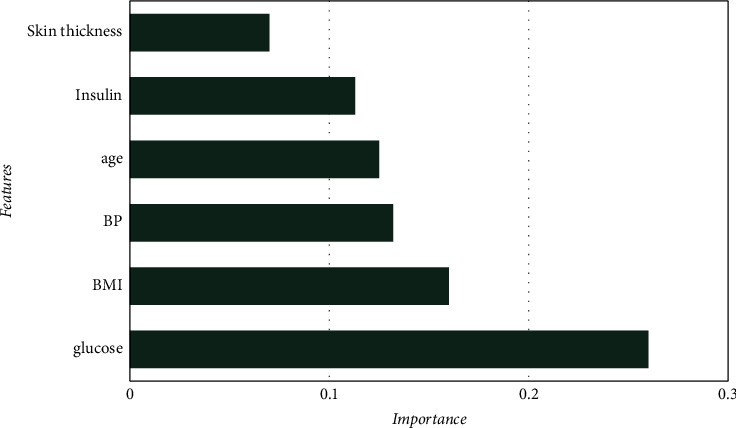
Important features.

Table 5 and Figure 4 represent that HBGB provided the highest accuracy, and the other performance metrics, such as precision (Figure 5), sensitivity or recall (Figure 6), and *F*1 score (Figure 7) are also very prominent for HBGB compared to other algorithms used in this study. For this method, the 5-fold cross-validation technique [27] and hyperparameter tuning technique were also applied. For the hyperparameter, the best max iter value was 100, and the best learning rate was found at 0.04. It is also seen that the DT model provides good accuracy as well. On the other hand, SVM provided the lowest accuracy in this method. As HBGB had shown the highest performance, this algorithm was selected to predict diabetes for our prediction system. Important features in Figure 8 are also determined to make the analytics graph based on these features [33].

### 3.1. Recommendations Based on User Input

#### 3.1.1. Patient Who Has Diabetes

If the diabetes prediction system indicates that the patient has diabetes according to the information provided, the CDSS will make recommendations based on this prediction. A sample of recommendations that will be displayed in the patient's user interface is given in Table 6 [34, 35].

#### 3.1.2. Patient Who Does Not Have Diabetes

**Figure 9:**
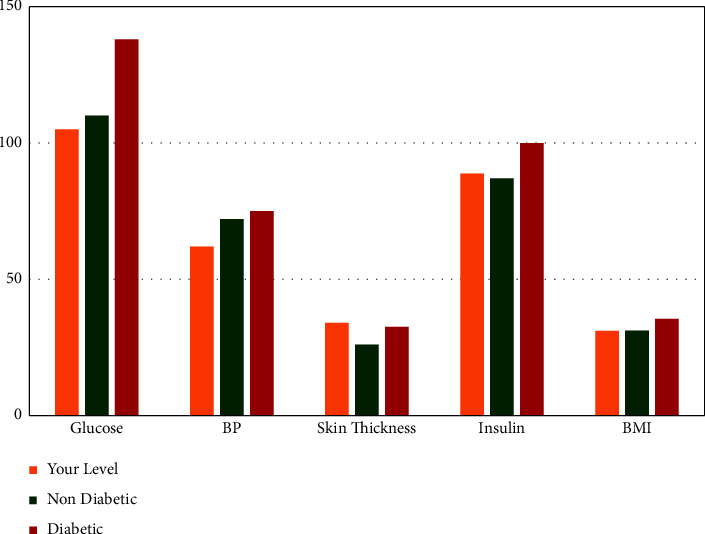
Comparative analytics for the nondiabetic patient.

On the other hand, patients who will be predicted negative for diabetes after checking their diabetes status by putting the required input fields on the user interface, as shown in Figure 3, will be relocated to another interface where the patient can perceive a comparative graph (Figure 9) of some critical parameters based on important features from Figure 8 such as BMI, glucose, BP, insulin level, and skin thickness. In Figure 9, it can be seen that the first level denotes the patients' current level parameter, which is obtained from their input value, the second level is the average of nondiabetic patients of the particular parameter, which is accumulated from the dataset, and finally, the third level is the average level of the diabetic patient's specific parameter. Thus, this will allow the patient to determine whether or not they are at risk for developing diabetes by analyzing different parameter levels from the graph. As a result, they will have a greater understanding of their current health status, allowing them to make more informed decisions, and it will also help them to learn better about chronic diabetes disease.

As can be seen, patients who are predicted as diabetic are given guidance, whereas those who do not have diabetes are also given comparisons of various input parameters. From this, the patient can be aware of different parameter levels and also can take necessary steps to control these particular levels like BMI, BP, and glucose. As a result, both diabetic and nondiabetic patients will be benefited from the proposed CDSS.

## 4. Conclusion

In this research, an expert system is presented to help physicians as well as patients to make decisions about diabetes diagnosis and to offer real-time analysis-based suggestions to improve medical quality. The time-consuming identification process leads to a patient's appointment at a diagnostic center and consultation with a doctor. For predicting diabetes, several ML classification techniques were applied with different scaling methods. A rule-based approach with the HBGB classifier provides the highest accuracy of 92.21%. Since the best result for our prediction system was obtained from HBGB, this algorithm can be used for the proposed CDSS. This proposed system will also be very beneficial for the nondiabetic patient as it will show a comparative analysis of different parameters that are directly responsible for diabetes disease. For the automation of diabetes analysis, the work can be expanded and enhanced. In future work, feature selection techniques can be implemented to check the effects on accuracy improvement with various subsets of features. It is also planned to collect data from many regions across the globe in the future to create a more accurate and broader predictive model for diabetes decisions. If daily data of diabetes patients can be put together, then a clinical support system can be implemented for daily decision support for patients worldwide.

## Figures and Tables

**Table 1:** Various parameters of different features of the dataset.

Feature name	Distinct	Distinct (%)	Minimum	Maximum	Zeros	Zeros (%)	Mean
BMI	248	32.3	0	67.1	11	1.4	31.99
Glucose	136	17.7	0	199	5	0.7	120.89
BP	47	6.1	0	122	35	4.6	69.10
Insulin	186	24.2	0	846	374	48.7	79.79

**Table 2:** Accuracy of different classification algorithms with the raw dataset.

Classification techniques	Accuracy (%)
K-nearest neighbor	76.62
Decision tree	78.02
Random forest	75.20
Support vector machine	77.27
Naïve Bayes	75.97
Histogram-based gradient boosting	79.88

**Table 3:** Accuracy of different classification algorithms after applying scaling methods.

Classifiers	Scaling methods					
	MinMax scaler (%)	Standard scaler (%)	MaxAbs scaler (%)	Robust scaler (%)	Quantile transformer (%)	Power transformer (%)
KNN	84.02	82.84	81.66	77.51	78.70	80.47
DT	75.15	75.15	75.15	74.56	73.96	73.96
NB	78.11	78.11	78.11	78.11	79.88	79.56
RF	78.11	77.51	82.25	78.70	77.51	78.42
SVM	77.51	76.92	79.29	79.88	77.51	76.90
HBGB	79.88	79.88	79.88	79.88	79.88	79.88

**Table 4:** List of the applied rules to the dataset.

Rules	Rule description
R1	IF BMI **<** 23.4 and DiabetesPedigreeFunction ≤0.647 THEN diabetes negative otherwise positive
R2	IF Glucose ≤100 and BloodPressure ≤70 THEN diabetes negative otherwise positive
R3	IF SkinThickness ≤22 and BMI **<** 25.8 THEN diabetes negative otherwise positive

**Table 5:** Different classification algorithms' performance metrics after applying the rule-based approach.

Classification techniques	Accuracy (%)	Precision (%)	Recall (%)	*F*1 score (%)
KNN	87.66	87.78	87.66	87.15
DT	91.56	91.52	91.56	91.53
SVM	71.39	68.85	70.78	69.27
RF	90.91	88.48	88.31	88.38
NB	77.27	76.38	77.27	76.53
HBGB	92.21	92.33	92.21	92.25

**Table 6:** Sample of recommendations for the patient with diabetes predicted.

Regular checking	Check glucose level and also meet with the doctor for further instruction.
Glycemic targets	Maintain blood glucose level before a meal: 80 to 130 mg/dL (4.4 to 7.2 mmol/L). Two hours after the start of a meal: Less than 180 mg/dL (10 mmol/L).
Follow doctor's prescriptions	Should follow the prescriptions of the doctors and take the medications on time and follow other instructions.
Exercise regularly	Aim for at least 150 minutes of moderate-intensity physical activity every week. Break it down into 30 minutes five times a week.
Avoid unhealthy carbohydrates	Eat less salt and be careful of red and processed meat. Drink alcohol sensibly and be smart with snacks and avoid smoking.
Choose healthier food	Start eating more fruit, vegetables, and dairy products like unsweetened yogurt and milk. We need energy, so choose healthier fats such as ghee, butter, lard, biscuits, cakes, pies, and pastries. It is still a good idea to cut down on using oils in general, so try to grill, steam, or bake foods instead.
Cut down on added sugar	Cutting out sugar can be really hard, so instead use low or zero-calorie sweeteners for beginning.

Source: World Health Organization (WHO), American Diabetes Association.

## Data Availability

The data used to support the findings of this study are available from the corresponding author upon request.

## Supplementary Materials

Supplementary Materials Figure S1: the graphical overview of the proposed system, where the whole system is represented using five stages.
