# Supplementary figures and images for: Clinical Decision Support System for Diabetic Patients by Predicting Type 2 Diabetes Using Machine Learning Algorithms
Source: J Healthc Eng. 2023 May 30;2023:6992441. doi: 10.1155/2023/6992441 (PMC10243956; doi:10.1155/2023/6992441)

**Graphical Overview of The System:**

**
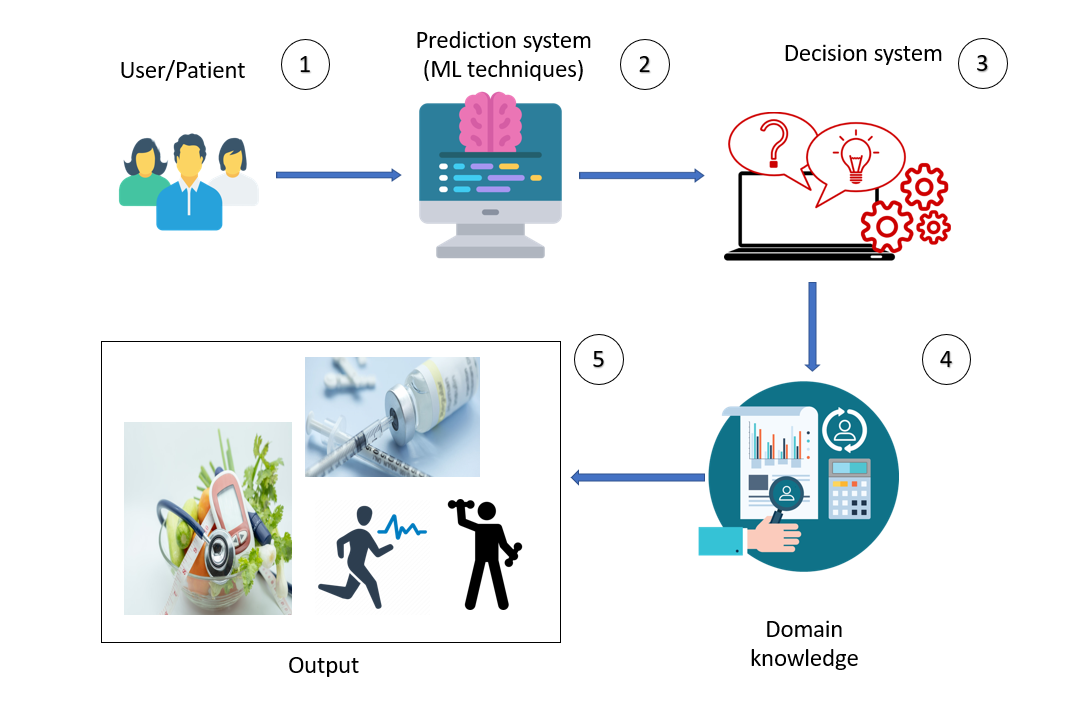
**

Figure S1: Graphical overview of the system.

Supplement: Supplementary Materials — Figure S1: the graphical overview of the proposed system, where the whole system is represented using five stages. [file 6992441.f1.docx]
